# Análisis farmacogenético retrospectivo de una paciente pediátrica en tratamiento anticoagulante: caso clínico

**DOI:** 10.7705/biomedica.5840

**Published:** 2021-09-22

**Authors:** Mirta Cavieres, Marcelo Suárez, Gabriel Verón, Luis Abel Quiñones, Nelson Miguel Varela

**Affiliations:** 1 Laboratorio Clínico, Hospital Dr. Luis Calvo Mackenna, Santiago, Chile Laboratorio Clínico, Hospital Dr. Luis Calvo Mackenna Santiago Chile; 2 Laboratorio de Carcinogénesis Química y Farmacogenética, Departamento de Oncología Básico-Clínico, Facultad de Medicina, Universidad de Chile, Santiago, Chile Universidad de Chile Departamento de Oncología Básico-Clínico Facultad de Medicina Universidad de Chile Santiago Chile; 3 Servicio de Farmacia, Hospital Clínico Red de Salud UC-Christus, Santiago, Chile Hospital Clínico Red de Salud UC-Christus Santiago Chile; 4 Red Latinoamericana para la Implementación y Validación de Guías Clínicas Farmacogenómicas (RELIVAF-CYTED), Santiago, Chile Red Latinoamericana para la Implementación y Validación de Guías Clínicas Farmacogenómicas (RELIVAF-CYTED) Santiago Chile

**Keywords:** acenocumarol, farmacogenética, anticoagulantes, vitamina K, Acenocoumarol, pharmacogenetics, anticoagulants, vitamin K

## Abstract

Se presenta el caso clínico de una paciente de 10 años diagnosticada con miocardiopatía dilatada, quien registró valores en el índice internacional normalizado *(International Normalized Ratio,* INR) superiores a 10 con la dosis estándar de acenocumarol, además de otros valores que indicaban el estado incoagulable, lo que obligó a suspender y reiniciar el tratamiento en varias ocasiones. Después de más de 30 días de tratamiento, sorprendentemente se lograron los niveles esperados y estables en el INR con la mitad de la dosis recomendada para una paciente de su edad y peso.

Se decidió hacer un análisis farmacogenético retrospectivo del caso mediante RT-PCR con sondas *TaqMan™* que incluyó cinco polimorfismos de un solo nucleótido y distinto grado de asociación con la dosis-respuesta a los fármacos antivitamínicos K (AVK): rs2108622 (gen *CYP4F2),* rs9923231, rs7294 (gen *VKORC1),* rs1799853 y rs1057910 (gen *CYP2C9).* La paciente resultó ser homocigota para el rs9923231 *(VKORC1)* y heterocigota para el rs2108622 *(CYP4F2).* Se ha evidenciado a nivel nacional e internacional que este perfil genético está fuertemente asociado con una necesidad de dosis menores de antivitamínicos K.

En conclusión, el análisis farmacogenético confirmó que la condición genética de la paciente, la cual conlleva una baja expresión de la enzima VKORC1 (blanco terapéutico de los antivitamínicos K), hacía predecible la necesidad de una dosis menor a la establecida según los protocolos clínicos recomendados por la *Food and Drug Administration* (FDA) y PharmGKB™ para los fármacos cumarínicos. El análisis genotípico previo de la paciente hubiese permitido alcanzar el rango terapéutico más prontamente, evitando potenciales riesgos de hemorragia, lo que demuestra la importancia de los análisis farmacogenéticos en tratamientos de gran variabilidad y estrecho rango terapéutico.

## Abstract (en)

We present the clinical case of a 10-year-old patient diagnosed with dilated cardiomyopathy who registered INR values above 10 upon receiving standard doses of acenocoumarol, as well as other values reported as uncoagulable, forcing the discontinuation and restart of treatment more than once. Expected and stable INR levels were achieved after more than 30 days of treatment, surprisingly with half the recommended dose for a patient of her age and weight. We decided to conduct a retrospective pharmacogenomic analysis including nucleotide genetic polymorphisms (SNPs) with different degrees of association with the dose/response to antivitamin K (AVK) drugs: rs2108622 (gene *CYP4F2),* rs9923231, rs7294 (gene *VKORC1),* rs1799853, and rs1057910 *(CYP2C9* gene) using TaqMan^®^ RT-PCR. The patient was homozygous for rs9923231 *(VKORC1)* and heterozygous for rs2108622 *(CYP4F2),* a genetic profile strongly associated with a requirement of lower AVK doses as shown by national and international evidence.

In conclusion, the pharmacogenetic analysis confirmed that this patient's genetic conditions, involving low expression of the VKA therapeutic target, required a lower dose than that established in clinical protocols as recommended by the *Food and Drug Administration* (FDA) and the PharmGKB^®^ for coumarin drugs. A previous genotypic analysis of the patient would have allowed reaching the therapeutic range sooner, thus avoiding potential bleeding risks. This shows the importance of pharmacogenetic analyses for highly variable treatments with a narrow therapeutic range.

Las enfermedades cardiovasculares son la principal causa de morbilidad y mortalidad en el mundo ^1^. Anualmente ocurren, aproximadamente, 17,5 millones de muertes por esta causa y se estima que, para el año 2030, la cifra aumentará a 23,6 millones, en especial, debido a enfermedades como fibrilación auricular, accidente cerebrovascular, infarto agudo de miocardio y miocardiopatías ^1^. Estas últimas son un grupo de enfermedades que afectan primariamente al músculo cardíaco e incluyen las valvulopatías adquiridas, la cardiopatía hipertensiva, la enfermedad coronaria o la pericárdica. Las miocardiopatías dilatadas de origen familiar se heredan por un mecanismo autosómico dominante ^2^^,^^3^. Entre los fármacos que se utilizan para su tratamiento, se encuentran los cumarínicos o antivitamínicos K (AVK) ^3^, los cuales tienen un estrecho margen terapéutico asociado a la variabilidad individual en la respuesta terapéutica, por lo que el riesgo de hemorragias puede aumentar durante el inicio del tratamiento debido a los ajustes en la dosis ^3^^,^^4^.

Los antivitamínicos K, como la warfarina y el acenocumarol, actúan inhibiendo competitivamente la vitamina K epóxido reductasa C1 (VKORC1), en tanto que, desde el punto de vista farmacocinético, los antivitamínicos K son metabolizados principalmente por el citocromo P450 2C9 (CYP2C9). Por otro lado, el CYP4F2 es el encargado de metabolizar la vitamina K hidroquinona a hidroxivitamina K, eliminándola del ciclo metabólico. En consecuencia, una disminución en su actividad aumenta la disponibilidad de vitamina K, lo que implicaría la necesidad de mayores dosis de antivitamínicos K ^5^.

Desde el punto de vista farmacogenético, la *Food and Drug Administration* (FDA) recomienda analizar tres polimorfismos de nucleótido simple *(Single-Nucleotide Polymorphism,* SNP) antes de administrar warfarina: el rs1799853, el rs1057910 (ambos en *CYP2C9)* y el rs9923231 *(VKORC1)*^6^; de este último se tiene el mayor nivel de evidencia ^7^^,^^8^ (cuadro 1). Por su parte, el *Clinical Pharmacogenetics Implementation Consortium* (CPIC) incluyó en su guía clínica del 2018 la eventual necesidad de analizar el rs2108622 *(CYP4F2)* para el tratamiento con warfarina ^9^. En un reciente estudio publicado por nuestro grupo en 304 pacientes adultos chilenos en tratamiento con acenocumarol, se estableció que las variables clínicas (edad, peso y sexo) y el INR inicial solo incidían en la variabilidad de la dosis en un 19 %, en tanto que las variantes genéticas rs9923231 *(VKORC1),* rs1799853 *(CYP2C9)* y rs1057910 *(CYP2C9)* daban cuenta de un 37 % de dicha variabilidad ^10^.

**Cuadro 1:** Resultado de los genotipos estudiados en la paciente, fenotipo esperado y recomendación de dosis para acenocumarol según la evidencia

Gen	*VKORC1*	*VKORC1*	*CYP2C9*	*CYP2C9*	*CYP4F2*
SNP	rs9923231	rs7294	rs1799853	rs1057910	rs2108622
Genotipo observado en la paciente	A/A	C/C	C/C	A/A	C/T
	Homocigoto para el SNP	Homocigoto wt	Homocigoto wt	Homocigoto wt	Heterocigoto
Fenotipo descrito para el genotipo observado^*^	Bajos niveles de la enzima VKOR	Bajo riesgo de hemorragias	Bajo riesgo de hemorragias	Bajo riesgo de hemorragias	Metabolismo intermedio de vitamina K hidroquinona
Recomendación de dosis para ACC^*^ según genotipo observado	Dosis menor de la estándar	Dosis estándar	Dosis estándar	Dosis estándar	Dosis estándar
Nivel de evidencia^*^	Alto	Bajo	Bajo	Moderado	Moderado

VKORC1: vitamina k epóxido reductasa c1; wt: *Wild Type* (alelo más frecuente); ACC: acenocumarol

^*^ Obtenido de https://www.pharmgkb.org ^14^

En este contexto, se presenta el caso clínico de una paciente de 10 años que no mejoró de la manera esperada con el protocolo de ajuste de dosis para el tratamiento con acenocumarol. Tras realizar los estudios farmacogenéticos, esto se explicó por su perfil genético y se correlacionó con las dosis de antivitamínicos K que finalmente lograron la mejoría de la paciente.

## Presentación del caso

Se trata de una paciente pediátrica con diagnóstico de miocardiopatía dilatada al nacer e insuficiencia mitral a los 9 años. El equipo de genética del Hospital Pediátrico "Dr. Luis Calvo Mackenna" confirmó que la causa de la miocardiopatía dilatada era genética, de tipo autosómica dominante. Dada la condición clínica de la paciente y por decisión del equipo médico, se inició el tratamiento con acenocumarol (jarabe de 1 mg/ml). En ese momento, la paciente tenía 10 años y su peso era de 29 kg. Antes del tratamiento con acenocumarol había recibido ácido acetilsalicílico (100 mg/día) durante dos meses. Al inicio del tratamiento con acenocumarol, la paciente recibió, además, los siguientes medicamentos: furosemida, captopril, digoxina e inmunoglobulina, ninguno con interacción con el acenocumarol (Micromedex™).

**Figura 1:**
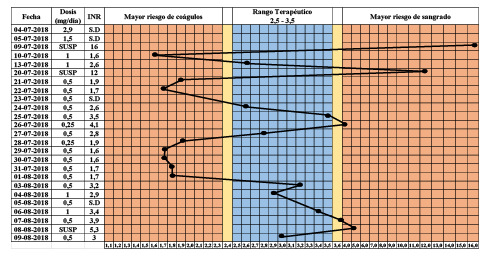
Valores del INR y dosis de acenocumarol administradas a la paciente (10 años de edad; 29 kg de peso) en los primeros 33 días de tratamiento

La dosificación de acenocumarol se ajustó a los protocolos del Hospital y a la "Guía práctica para el tratamiento anticoagulante en pediatría" de la Sociedad Chilena de Hematología basada en estudios nacionales e internacionales ^11^. La administración del acenocumarol comenzó con una dosis de carga de 0,1 mg/kg (2,9 mg/día) y una dosis diaria de 0,05 mg/kg (1,5 mg/día), con lo cual el valor esperado de INR debía haber estado entre 2,5 y 3,5 ^12^^,^^13^, según las pautas de tratamiento del Hospital. Cinco días después de iniciado el tratamiento, dicho valor era de 16,0 (incoagulable), razón por la cual se administraron 10 mg de vitamina K (por vía oral) y se suspendió el anticoagulante por ese día. Al día siguiente, el valor de INR fue de 1,6. Posteriormente, la dosis de mantenimiento se ajustó a 1 mg/día y el INR arrojó un valor de 2,6 (figura 1).

Fecha (DD-MM-AAAA), correspondiente al día en que fue tomada la muestra; INR: *International Normalized Ratio;* SUSP: dosis suspendida; SD: sin datos Nota: la fecha 04-07-2018 corresponde al día en que se administró la dosis de carga inicial de acenocumarol.

Siete días después, la paciente ingresó al servicio de urgencias del Hospital con un INR de 12,0. Se ajustó nuevamente la dosis a 0,017 mg/ kg (0,5 mg/día) y, cuatro días después, la paciente alcanzó un INR de 4,1. Los posteriores ajustes consecutivos permitieron alcanzar un INR de 2,3. En la figura 1, se presenta en detalle la dosificación y los valores de INR de la paciente hasta los 33 días de iniciado el tratamiento con acenocumarol.

El 9 de agosto del 2018, la paciente fue trasladada a otro centro asistencial de salud y no se pudo continuar su seguimiento.

Los últimos datos obtenidos datan de agosto del 2019, cuando la paciente asistió a control en el Hospital. En esa fecha, tenía 11 años de edad, pesaba 49 kg, y recibía una dosis de acenocumarol de 0,02 mg/kg (1 mg/día), la mitad de la recomendada para pacientes con esa edad y peso ^11^. Sin embrago, su INR se mantenía dentro del rango terapéutico ^2^^,^^5^^-^^3^^,^^5^.

### Análisis genético

Para la identificación de los SNP rs2108622 (gen *CYP4F2),* rs9923231, rs7294 (ambos en el gen *VKORC1),* rs1799853 y rs1057910 (los dos en el gen *CYP2C9),* se obtuvo ADN genómico de la paciente a partir de una muestra de sangre venosa periférica, utilizando un estuche comercial (EZNA Blood DNA minikit™ (Omega Bio-tek). La determinación de los genotipos estudiados (cuadro 2) se hizo por discriminación alélica mediante sondas TaqMan™ y PCR en tiempo real. Los reactivos utilizados fueron TaqMan Genotyping Master Mix™ y TaqMan Drug Metabolism Genotyping™ Assay (ThermoFisher Scientific, USA).

**Cuadro 2:** Genes analizados y secuencias contexto

Gen (SNP)	Tipo	Secuencia contexto	ID del ensayo
*VKORC1*	wt	GATTATAGGCGTGAGCCACCGCACC[C]GGCCAATGGTTGTTTTTCAGGTCTT	C__305840261_20
(rs9923231)	MAF	GATTATAGGCGTGAGCCACCGCACC[T]GGCCAATGGTTGTTTTTCAGGTCTT	
*VKORC1*	wt	GGCACATTTGGTCCATTGTCATGTG[C]GGGTATGGCAGGAGGAGGGGGTAAT	C___7473918_10
(rs7294)	MAF	GGCACATTTGGTCCATTGTCATGTG[T]GGGTATGGCAGGAGGAGGGGGTAAT	
*CYP4F2*	wt	CCCCGCACCTCAGGGTCCGGCCACA[C]AGCTGGGTTGTGATGGGTTCCGAAA	C__16179493_40
(rs2108622)	MAF	CCCCGCACCTCAGGGTCCGGCCACA[T]AGCTGGGTTGTGATGGGTTCCGAAA	
*CYP2C9*	wt	GATGGGGAAGAGGAGCATTGAGGAC[C]GTGTTCAAGAGGAAGCCCGCTGCCT	C__25625805_10
(rs1799853)	MAF	GATGGGGAAGAGGAGCATTGAGGAC[T]GTGTTCAAGAGGAAGCCCGCTGCCT	
*CYP2C9*	wt	TGTGGTGCACGAGGTCCAGAGATAC[A]TTGACCTTCTCCCCACCAGCCTGCC	
(rs1057910)	MAF	TGTGGTGCACGAGGTCCAGAGATAC[C]TTGACCTTCTCCCCACCAGCCTGCC	C__2710492_10

wt: *Wild Type* (alelo más frecuente); MAF: *Minor Allele Frequency* (alelo menos frecuente); [N]: cambio nucleotídlco que da cuenta del polimorfismo de un solo nucleótldo o SNP. Extraído de TaqMan® SNP Genotyplng Assays (Número de catálogo: 4362691), ThermoFlsher Scientific®

Los resultados y la interpretación de los análisis genéticos se presentan en el cuadro 1, los cuales se recibieron el 9 de agosto del 2018, informando que la paciente era homocigota para el rs9923231 *(VKORC1* ) y heterocigota para el rs2108622 *(CYP4F2).* Este perfil genético implica que las dosis de cumarínicos deben ser menores que las establecidas según criterios clínicos para alcanzar el rango terapéutico deseado (en este caso, un INR entre 2,5 y 3,5).

### Aspectos éticos

Para la publicación de este caso clínico, se contó con el consentimiento informado firmado por la madre de la paciente y la autorización del Comité de Ética Científico Pediátrico del Servicio de Salud Metropolitano Oriente del Ministerio de Salud, Chile.

## Discusión

Los polimorfismos genéticos y sus efectos en la dosis de los fármacos, entre ellos los antivitamínicos K, han sido estudiados ampliamente ^14^. Se han desarrollado diversos algoritmos de dosificación, principalmente para el uso de la warfarina en diferentes etnias, utilizando variables clínicas y farmacogenéticas, con el fin de mejorar la seguridad (evitar el riesgo de hemorragia) y la eficacia (disminuir el tiempo para el ajuste de la dosis) del tratamiento.

El presente caso clínico corresponde a una paciente pediátrica que no respondió de manera adecuada a la dosis estándar de acenocumarol, lo que obligó a constantes modificaciones y ajustes en la pauta de dosificación durante más de 30 días (figura 1). El análisis genético de la paciente evidenció un fenotipo metabolizador normal de la vitamina K hidroquinona (heterocigota para rs2108622, *CYP4F2),* pero una baja expresión de la VKORC1, enzima blanco de este fármaco (homocigota para rs9923231) (cuadro 1). El rs9923231 está localizado en la región promotora del gen *VKORC1* y el cambio nucleotídico (NC_000016.10:g.31096368C>T) reduce la unión de factores de transcripción, desencadenando una disminución en la expresión del mRNA, y con ello, también en la cantidad de la enzima VKORC1 (13). Por otro lado, el SNP rs2108622 (NC_000019.10:g.15879621C>T) produce un cambio en la secuencia de aminoácidos de la CYP4F2 (NP_001073.3:p.Val433Met), reduciendo su actividad. No obstante, en estudios internacionales se ha evidenciado que su efecto es recesivo, por lo que la condición heterocigota en la paciente no implicaría un cambio fenotípico significativo ni se asociaría con la necesidad de modificar la dosis estándar de antivitamínicos K. Es importante señalar que la frecuencia de estos SNP (rs9923231 y rs2108622) en la población chilena es alta: 0,467 y 0,229, respectivamente ^10^.

El perfil genético observado explicaría los altos valores del INR que la paciente presentó frente a la dosis estándar de acenocumarol, para la que solo se consideraron variables clínicas (principalmente antropométricas). Cabe destacar que la paciente alcanzó cuatro veces una condición de anticoagulación excesiva y, por ende, un gran riesgo de hemorragia (figura 1). Los últimos registros de la paciente (agosto de 2019) muestran que logró alcanzar el rango terapéutico (INR entre 2,5 y 3,5) con la mitad de la dosis recomendada de acenocumarol. De haber tenido los análisis farmacogenéticos aquí descritos en el momento de iniciar el tratamiento con este fármaco, se hubiera podido alcanzar el rango terapéutico en menos tiempo (mayor eficacia) y mejorar sustancialmente la seguridad del tratamiento (evitando episodios de INR sobre 3,5).

## Conclusión

Los resultados obtenidos confirman que la paciente presentaba un fenotipo farmacocinético y farmacodinámico que requería dosis de antivitamínicos K menores a las establecidas en los protocolos que consideran únicamente variables antropométricas, tal y como se ha establecido en estudios internacionales y nacionales previos. Es necesario implementar criterios farmacogenéticos en las guías clínicas del tratamiento anticoagulante con antivitamínicos K, siguiendo las recomendaciones de organismos regulatorios como la FDA dada la gran variabilidad observada en la reacción a los antivitamínicos K, su estrecho margen terapéutico y la frecuencia con que estos polimorfismos genéticos se presentan en nuestra población. La incorporación de variables respaldadas por evidencia sólidas en los algoritmos de dosificación permitiría alcanzar una mayor seguridad y eficacia del tratamiento anticoagulante.

## References

[1] Organización Mundial de la Salud Enfermedades cardiovasculares.

[2] Vukasovic JL (2015). Miocardiopatía dilatada: aspectos genéticos, infecciosos, inflamatorios y del sistema inmune. Revista Médica Clínica Las Condes.

[3] Landefeld CS, Beyth RJ (1993). Anticoagulant-related bleeding: Clinical epidemiology, prediction, and prevention. Am J Med.

[4] White HD, Gruber M, Feyzi J, Kaatz S, Tse HF, Husted S (2007). Comparison of outcomes among patients randomized to warfarin therapy according to anticoagulant control: Results SPORTIF III and V. Arch Intern Med.

[5] Florez J, Sedano MC (2014). Farmacología humana: farmacología de la hemostasia, la coagulación y la fibrinólisis.

[6] U.S. Food and Drug Administration Table of pharmacogenomic biomarkers in drug labeling.

[7] Whirl-Carrillo M, McDonagh EM, Hebert JM, Gong L, Sangkuhl K, Thorn CF (2012). Pharmacogenomics knowledge for personalized medicine. Clin Pharmacol Ther.

[8] Owen RP, Gong L, Hersh S, Klein TE, Altman RB. (2011). VKORC1 pharmacogenomics summary. Pharmacogenet Genomics.

[9] Johnson JA, Caudle KE, Gong L, Stein CM, Scott SA, Lee MT (2017). Clinical Pharmacogenetics Implementation Consortium (CPIC) Guideline for pharmacogenetics-guided warfarin dosing : 2017 update. Clin Pharmacol Ther.

[10] Roco A, Nieto E, Suárez M, Rojo M, Bertoglia MP, Verón G (2020). A pharmacogenetically guided acenocoumarol dosing algorithm for Chilean patients: A discovery cohort study. Front Pharmacol.

[11] Bonduel M, Sciuccati G, Hepner M, Feliu-Torres A, Pieroni G, Frontroth JP (2003). Acenocoumarol therapy in pediatric patients. J Thromb Haemost.

[12] Holbrook A, Schulman S, Witt DM, Vandvik PO, Fish J, Kovacs MJ (2012). Evidence-based management of anticoagulant therapy: Antithrombotic therapy and prevention of thrombosis, 9th ed: American College of Chest Physicians Evidence-Based Clinical Practice Guidelines. Chest.

[13] Guyatt GH, Akl EA, Crowther M, Gutterman DD, Schünemann HJ (2012). Executive summary: Antithrombotic therapy and prevention of thrombosis. 9th edition. American College of Chest Physicians Evidence-Based Clinical Practice Guidelines. Chest.

[14] Potamias G, Lakiotaki K, Katsila T, Lee MTM, Topouzis S, Cooper DN (2014). Deciphering next-generation pharmacogenomics: An information technology perspective. Open Biol.

